# Advancing Ethics and Policy for Healthy‐Volunteer Research through a Model‐Organism Framework

**DOI:** 10.1002/eahr.500001

**Published:** 2019-01-22

**Authors:** Jill A. Fisher, Rebecca L. Walker

**Affiliations:** ^1^ Associate professor in the Department of Social Medicine and the Center for Bioethics at the University of North Carolina at Chapel Hill; ^2^ Professor in the Department of Social Medicine and the Center for Bioethics at the University of North Carolina at Chapel Hill.

**Keywords:** human research ethics, phase I clinical trials, healthy‐volunteer research, animal research ethics, research oversight

## Abstract

Nonhuman animal research and phase I healthy‐volunteer clinical trials are both critical components of testing the safety of investigational drugs as part of the development of new pharmaceuticals. In addition, these types of research share important structural features, as both take place in confinement and both use subjects that are dissimilar to the target population. By mobilizing a *model‐organism* framework for phase I trials, we employ concepts and mechanisms typical to animal research to query gaps in the human subjects ethics and policy framework. By bringing these two research worlds together, we aim to illustrate how the model‐organism framework can enhance healthy volunteers’ welfare during trials, improve research oversight, and more critically assess the science value of current phase I trials.

**N**onhuman animal research (hereinafter “animal research”) and phase I clinical trials are closely connected in the research pipeline for drug development. Preclinical animal studies of investigational drugs aim to better gauge the safety of drugs before exposing human subjects to them and may also look for evidence of potential efficacy in animal models for the targeted human condition. Phase I trials test the safety and tolerability of investigational drugs and are typically conducted on healthy volunteers who are exposed to some risk but derive no direct medical benefits from their participation. Beyond emphasis on testing drug safety, preclinical animal studies and phase I trials share important structural features, as both take place in confinement and both involve research subjects that are dissimilar to the target population. These structural features of the research may also lead to similar challenges both of validity and translation. Yet, despite the commonalities, preclinical animal studies and phase I trials are rarely discussed together. Moreover, the two types of research are covered by entirely different sets of regulatory standards and oversight mechanisms.

Recently, some bioethics scholars have asked whether ethical principles for research with humans—for example, regarding children’s and adolescents’ assent to or dissent from participation—should apply to research with some animals.^1^ That work focuses on a need for ethical standards that reflect the shared capacities of human and animal research subjects. We suggest, however, that there is parallel ethics and policy work that can be done in considering the shared structural features of phase I healthy‐volunteer trials in particular and preclinical biomedical research with animals. Further, we think the most useful comparative work in this arena is in flipping the application—that is, in applying animal research concepts and oversight mechanisms to phase I clinical trials. In short, we propose considering the implications of a *model‐organism* approach to healthy‐volunteer trials.

In animal research, the term “model organism” refers to “organisms on which much is known, and knowledge of which can be freely and easily accessed and used to study other organisms.”^2^ Animals are selected based on their suitability to answer particular types of research questions in biomedicine, their presumed shared characteristics with and the expectation that information about them can be extrapolated to humans, and/or their availability and the ease of procuring them.^3^ In the case of phase I trials, healthy volunteers are patently not different “organisms” than members of the average patient population. Such a claim would be absurd. However, phase I participants do stand in for the target population *for any drug* regardless of the therapeutic area while themselves meeting narrow health parameters and typically participating serially in studies.^4^ The controlled research environment adds another layer to the model given that participants are subject to standardized diets and have restrictions placed on their physical activity.

Considering humans as model organisms is ethically fraught, and we must therefore be absolutely clear that we neither imply any moral status denigration of human subjects nor endorse the ethical appropriateness of the animal research oversight structure as a whole. Indeed, there are significant ethical concerns to be had about particular aspects of the regulatory structure for research with animals.^5^ Our interest, instead, is in using concepts and mechanisms typical to animal research to query gaps in the ethics and policy framework for research with humans as these arise specifically for healthy‐volunteer trials. In what follows, we first examine these gaps as they are related to structural features of phase I participation. We then consider the concepts from and mechanisms of the animal research ethics and policy structure that are particularly relevant to phase I trials. Finally, we bring these worlds together in offering some specific points of intersection to improve the ethics and policy approach to phase I trials. As we will demonstrate, applying the model‐organism framework to phase I trials productively challenges the translational science value of the use of healthy volunteers while also offering novel insights into the ethical issues that are endemic to this type of research.

## STRUCTURAL FEATURES OF PHASE I TRIALS AND GAPS IN ETHICS OVERSIGHT

**I**n spite of the unique structural features of healthy‐volunteer clinical trials, there are no special ethics or policy approaches to them. As with all human subjects research in the United States, phase I trials must adhere to ethical standards established as part of the *Belmont Report* and codified in federal regulations. The three ethical pillars of human subjects research were identified as respect for persons, beneficence, and justice, which were operationalized respectively through the processes of informed consent, risk‐benefit assessment, and the appropriate selection of research participants. While each of these approaches to protecting research participants is essential for healthy‐volunteer trials, we will show how there are ethics and policy gaps that are important to resolve in each ethical domain that results from the confinement of healthy volunteers during the course of a study and from monetary compensation for their participation.

Unlike with later‐phase clinical trials, a critical structural feature of phase I healthy‐volunteer trials is that they typically include a period during which participants are not permitted to leave the facility unless they withdraw from the study. The confinement can last weeks and is designed to ensure that all trial participants consume the same foods and beverages, abstain from prohibited activities, and are available for frequent procedures, such as blood collection, for data about the investigational drug. As with animal research, the controls put into place in the phase I research environment are considered critical to valid and generalizable studies.

A second important structural feature of phase I trials is that healthy volunteers are recruited with the promise of sizeable stipends, ranging from $100 to $300 per day.^6^ The combination of the lengthy confinement and the opportunity to earn income in this way means that unemployed or underemployed people often enroll in these studies. Moreover, these studies typically attract economically disadvantaged minority men, especially Latinos and African Americans.^7^ With limited opportunities to earn stable income, many such healthy volunteers participate repeatedly in new studies, with some even treating phase I trial participation as full‐time work.^8^ While serial enrollment and financial incentives make these participants readily available for studies, the small pool of predominantly male healthy individuals on whom most phase I trials are conducted raises critical questions about study validity and translation similar to those that arise with animal research. At stake are not only science‐value questions about how resulting safety and tolerability data can be extrapolated and applied to affected patient populations but also more traditional ethical and policy challenges that come with phase I research, such as financial inducement and fairness in the distribution of risks.

To meet the ethical principle of respect for persons, the informed consent process bears significant burden in resolving many of the ethical problems inherent to human subjects research. If prospective participants with the capacity to understand and provide consent have been thoroughly informed about the nature of the research, alternatives, and the right to withdraw, a significant aspect of the ethical duty of the researcher has been met. Remaining ethical concerns are generally that those participants could have been unduly induced or coerced into consenting. Yet conventional approaches to appraising undue inducement and coercion seem to have major limitations when the research relationship, as it is with healthy‐volunteer trials, is based primarily on a financial transaction. First, because the compensation motivates healthy volunteers to enroll, it is much more difficult to define how much money creates an undue inducement. Healthy volunteers themselves sometimes reject the logic of undue inducement as a convenient fiction of drug companies in whose interest it is to undercompensate, and yet they also readily admit to participating in studies in some sense over their own objection due to the amount of compensation offered.^9^ Specifically, some healthy volunteers have voiced reservations about what they see as unacceptable risks of participation but nonetheless feel that the money offered is too good to refuse.^10^ Second, in spite of frequent conflation between coercion and undue inducement in research oversight,^11^ typical bioethical understandings of coercion insist that, unlike with undue inducement, genuine offers cannot coerce. Nonetheless, the socioeconomic conditions in which healthy volunteers choose to participate in clinical trials may evidence material threats to their well‐being, such as those characterized by economic insecurity, race‐based employment discrimination, histories of incarceration, and/or undocumented immigration. Elsewhere, we have described this consent context as potentially one of structural coercion that compels certain groups in society to enroll in research.^12^

Likewise, the ethical principle of beneficence is insufficiently honed to address the peculiarities of healthy‐volunteer trials. Achieving beneficence requires researchers to minimize the risk of harm to participants and offset any remaining risk by the potential for individuals or society to benefit from the research. Even when risks are minimized, phase I trials are distinct from later‐phase trials in that the goal of this research is precisely to induce harm in healthy volunteers to generate data about the safety and tolerability of investigational drugs. Meta‐analyses of healthy‐volunteer trials indicate that they are relatively safe overall, meaning that most harms that occur are relatively mild and short term.^13^ Regarding risk of serious harm, very little might be known in the case of first‐in‐human trials about the risks of an investigational agent, but despite precautions to begin with doses that are expected to be nearly pharmacologically inert,^14^ tragedies can occur.^15^ Moreover, little is known about the cumulative risk of serial participation, and it is impossible to account for the potential of cumulative risk in a conventional study‐by‐study mode of risk assessment.

In addition, the standard ethical balancing of risk and potential for benefit does not fully encapsulate the experience of healthy volunteers. While not all participants develop adverse effects (that is, the side effects about which information is sought for investigational drugs), frequent blood draws and other forms of monitoring are universal experiences that may be insufficiently attended to in the risk‐benefit analysis. Further, for many healthy volunteers, being subjected to confinement and activity restrictions not only is onerous but can also be recast as a harm of participation, as we discuss in more detail below. Overall, because the financial benefit that motivates their participation cannot itself be seen to offset health risks or experienced harms,^16^ trial risks are said to be ethically justified by the resulting societal benefit. While affected patients who enroll in clinical trials might either benefit individually or identify more readily with those future patients with the same disease or illness who are benefited, such societal benefit is much more of an abstract concept for healthy volunteers.

The structural features of healthy‐volunteer studies also have important implications for the ethical principle of justice. Typically, concerns about justice in research encourage attention to the fair selection of participants as well as fair allocation of the benefits and risks of research participation. In later‐phase trials, a frequent concern centers on the underrepresentation of racial minorities. Phase I trials, however, turn this concern on its head because minorities are actually *over*represented in terms of sheer numbers as well as relative to the population as a whole.^17^ Given that there is no possible medical benefit to healthy volunteers, exposing minority groups to the risks and harms—even if minimal—of phase I trials might be exploitative rather than inclusive, especially when taking into account disproportionate economic disadvantages that make healthy volunteers, who frequently lack health insurance, unlikely to benefit from the drug discovery process. These features of phase I trials suggest that the pharmaceutical industry may place an unfair burden on minority groups to test the safety of their products.

In addition to upholding the principles of respect for persons, beneficence, and justice, procedural norms and science value are critical to an adequate ethics and policy framework for human subjects research.^18^ Procedural norms and science value, as traditionally operationalized, also have limitations given the structural features of healthy‐volunteer clinical trials. Procedural ethics is most evident in the standard for independent review of research protocols that is the responsibility of institutional review boards (IRBs). However, IRBs are focused on protocol review, and as we will demonstrate below, many of the ethical lapses in phase I research occur at clinical trial sites themselves, which are not closely monitored. Further, while research that has limited scientific value is thereby ethically questionable, science‐value problems for phase I research are little recognized. As we shall explain, taking seriously the model‐organism framework for healthy volunteers gives insight into how oversight mechanisms and science‐value assumptions must be renegotiated to adequately protect healthy volunteers. To make this comparison, we must first examine conventional ethics and policy approaches to nonhuman animal research.

## ETHICS AND POLICY APPROACHES TO BIOMEDICAL ANIMAL RESEARCH

**H**uman and animal research programs operate under different ethics and policy frameworks.^19^ Unlike human subjects research, with its guiding ethical principles of respect for persons, beneficence, and justice, animal research has a circumscribed concern for animal welfare and the replacement, reduction, and refinement of animal use.^20^ We describe these aspects of the ethics and policy framework for research with animals to set the stage for how they might be productively extrapolated to the phase I human trial context.

The principle of welfare in animal research attends to the quality of life of laboratory animals. Their housing, including its cleanliness and size, and their environment more generally, such as interactions with each other and ability to engage in normal behaviors, are among the quality‐of‐life concerns.^21^ In addition, researchers

**The confinement and monitoring of healthy volunteers in phase I clinical trials invite questions of how to regulate the research environment, respectfully engage with participants, and provide for their comfort and welfare.**

are obligated to minimize stress that laboratory animals might experience and ensure that the animals are healthy or receive appropriate veterinary care. Finally, researchers are expected to not subject animals to unnecessary pain and suffering beyond the specific ends of the scientific protocol; for example, researchers should use anesthesia and analgesics to minimize pain caused by procedures and euthanize animals whose suffering cannot otherwise be controlled.^22^ Concerns about animal welfare, however, do not preclude the serious harm that routinely occurs to animals in research both as a reality of the scientific goals of research and due to confinement in a research facility. An important part of upholding animal welfare, recognized in European Union Directive 2010/63, is nonetheless balancing the harms to animals and the potential for human benefit of individual research protocols.

In addition to the guiding principle of animal welfare and the related ethical requirement for harm‐benefit analysis, there are the so‐called 3Rs of animal research—replace, reduce, refine—which were developed as a professional ethics framework in the mid‐twentieth century by two scientists concerned about gaps in the humane treatment of laboratory animals.^23^ Today, these concepts are incorporated into regulatory and policy guidance for animal research both nationally and internationally. In brief, the concept of replacement encourages alternatives to the use of live animals in research whenever feasible, such as with in vitro studies or computer simulations. Other than avoiding the use of animals altogether in research, relative replacement also encourages the use of less‐sentient creatures, such as some invertebrates, to minimize pain and distress that research may cause.^24^ When replacement of live animals is not possible, researchers are encouraged to follow the principle of reduction, meaning to use the smallest number of animals needed for the aims of the research or to maximize data collection from each animal and protocol while not increasing pain or distress. Similarly, the principle of refinement advocates for researchers to alter the ways research protocols are designed so that experimental subjects are exposed to less pain and suffering over the course of the research. Refinement can also include improvements to animals’ housing and the addition of enrichment measures to improve the quality of their lives. While animal advocates tend to focus solely on replacement, whereas animal research oversight committees tend to emphasize refinement, together the 3Rs underscore that the use of animals in research should be undertaken with great care and that it is the duty of researchers involved in such work to promote the broader principle of welfare.

As with human subjects research, there is a system of procedural ethics and science‐value assessment that occurs in the oversight of animal research. In the United States, both the Animal Welfare Act and the Public Health Service’s policies for animal research require oversight and approval of animal research by an institutional animal care and use committee (IACUC). An IACUC may suggest changes to procedures, monitoring, or pain‐control regimes or may require a better justification of research with animals or of the numbers used in a study. IACUCs also conduct routine inspections of all the animal facilities and laboratories under their purview; provide a general review of the institutional research program, including offering suggestions about researcher and technician training; and address ad hoc animal‐welfare concerns that arise. These IACUC functions aim to ensure that animal welfare and the 3Rs are at the forefront of institutions’ orientation to research with laboratory animals.

In animal research generally, a critical assessment of science value is an explicit concern as researchers attend carefully to the translation of animal research findings for use in research on humans, such as clinical trials. In selecting and justifying the animals chosen for specific biomedical research programs, researchers must balance different types of validity, including face validity (how well the species is able to model the clinical condition itself), construct validity (how well the species elucidates the etiological or physiological processes underlying a condition), and predictive validity (how well the species’ results translate to a clinical, human context).^25^ Although there are limitations to how well science‐value assessments are done, attention to—and anxiety about—translation and reproducibility underscore the importance of these concepts in animal research.^26^

## MODEL‐ORGANISM APPROACH TO HEALTHY‐VOLUNTEER RESEARCH ETHICS & POLICY

**R**econsidering the ethics gaps in healthy‐volunteer trials, a model‐organism approach capitalizes on shared structural features between phase I clinical trials and animal research to examine in a new way the seemingly intractable human subjects regulatory and policy problems outlined above. It can be said that healthy volunteers are valuable to phase I research because they are a type of model organism. Within the biomedical sciences, the use of model organisms provides standardization and predictability to research arenas. For example, using model organisms allows for a type of common language among researchers, creating the possibility for the exchange and comparison of scientific results as well as shared assumptions about how results can be extrapolated from the model organism to humans. The predictability of the model also aids the research process because investigators know what to expect from their animal subjects and this knowledge can help with the interpretation of their findings.^27^

Healthy volunteers are similarly helpful in phase I trials, as they approximate the role of model organisms in other research contexts. Healthy volunteers create a basis for common trial protocols across different therapeutic areas to establish standardized measures of drug safety and tolerability. Unlike ill patients, healthy volunteers provide a baseline to evaluate the effects of an investigational drug without the “noise” that an underlying disease might create in the data. Because phase I trials take place in controlled environments and with regimented diets and activity restrictions, the comparison with model organisms is strikingly apt for the healthy‐volunteer population.

Yet, in spite of the structural similarities between healthy‐volunteer trials and animal research, there has been little attention to probing what this might mean for how phase I trials might need different ethics and policy approaches than many later‐phase trials. In this section, we will describe the implications of the model‐organism framework for enhancing healthy volunteers’ welfare during trials, improving research oversight, and more critically assessing the science value of current phase I trials.

### Welfare concerns

While typically used in the context of animal research, welfare is, of course, a concept routinely applied to humans to refer to structures that can promote their well‐being in everyday life. The model‐organism framework for phase I research directs focus to the welfare concerns that accompany the long‐term confinement of healthy volunteers to a research facility.^28^ Acknowledging the limitations on welfare promotion in the animal research case, we nonetheless can bring the concept, and the structural practices meant to promote it, to bear in the case of phase I research. Through the lens of the 3Rs, we will illustrate how welfare issues manifest for healthy volunteers particularly in terms of housing and study procedures as well as the appropriate use and number of healthy volunteers in phase I research. Further, considering harm‐benefit analyses under the umbrella of welfare, we are able to draw attention to a balancing, not merely of risks of harm, but actual harms, including non‐drug‐related ones, that are a routine part of clinical trial participation.

Clinics that conduct phase I trials are not designed in a standardized way, and, therefore, they do not all promote participants’ welfare equally. Some clinics are located within academic medical centers, whereas others are freestanding buildings owned and operated by commercial enterprises, such as contract research organizations. These differences matter in terms of how the space is configured and how many participants can be housed. For example, some facilities have semiprivate rooms for participants that resemble—or, in fact, were—hospital rooms with two or three beds. Other facilities use bunk beds to maximize their space in dormitory‐like configurations, but this can result in having more than a dozen participants in one room. Our prior research suggests that these latter clinic configurations can result in overcrowding, and there are no external standards on how many participants can share the space.^29^

Moderating the housing conditions are other factors, such as the comfort level of beds, chairs, and other furniture; access to windows; ambient room temperature; and poor‐quality food.^30^ Healthy volunteers routinely complain about how some clinics have thin mattresses, broken or inadequate furniture, windowless spaces, and distressingly cold rooms. Bracketing the diet parameters dictated by study protocols, healthy volunteers voice concern that the meals are of very poor quality and without sufficient variety. Even if participants were expected to spend only 24 hours in such spaces, these conditions could be quite unpleasant, but the fact that they often must remain in these facilities for days or weeks at a time makes it all the more problematic that their welfare is not always a priority. Extrapolating from the concept of refinement in animal research, these problems in healthy‐volunteer trials indicate that appropriate standards should be developed, implemented, and enforced to ensure that phase I clinics are adequate, if not comfortable, spaces for participation.

Another aspect of participant welfare to which the concept of refinement is germane is the pain and distress that frequent blood collection can cause for healthy volunteers. On days during which participants receive a dose of the investigational drug, data for the trial normally include a series of 10 to 12 blood collections over the course of several hours. Most clinics take blood by performing venipuncture each time a sample is needed (the “straight stick” method), but many participants greatly prefer when a peripheral venous catheter is placed to enable access to blood throughout the day and avoid repeated venipuncture. Adding to bruising and pain that can occur, many clinics hire staff members who are untrained or unskilled in phlebotomy and thus need to make multiple attempts before successfully collecting blood and may even rupture a vein in the process. Apart from experiencing immediate discomfort, healthy volunteers, especially those who enroll serially in trials, may be permanently scarred. To minimize pain and long‐term damage to participants, a welfare orientation would indicate that only staff members proficient at performing venipuncture should collect participants’ blood and that the protocol dictate the collection technique that is in the best interest of the participant, not the clinic.

Finally, in light of the 3R concepts of replacement and reduction, the use of healthy volunteers might be more pervasive in phase I testing than is ethically justified. Specifically, phase I trials often have relatively narrow scientific goals, utilizing different groups of healthy volunteers to test an investigational drug in a single dose and multiple doses; to compare different modes of administration or formulations (such as tablet versus capsule); to measure the effects of food on the action of the drug; to investigate the drug’s cardiac, hepatic, or renal effects; and to investigate the drug’s interaction with other, commonly prescribed drugs. As a result, hundreds of healthy volunteers might be used in dozens of separate phase I trials.^31^ The concept of replacement requires reflection on the necessity of using healthy volunteers for each of these scientific goals. Should high‐quality data be accessible, for example, through later‐phase clinical trials on affected patients, this would indicate that healthy volunteers should not be used for that particular area of scientific assessment. In trials that necessitate healthy volunteers, the concept of reduction would encourage researchers to maximize the data they collect in each phase I protocol and from each healthy volunteer. However, as we also learn from the 3Rs, attention must be paid to potential conflicts between reduction and refinement when maximizing data collection from individuals.^32^

### Procedural ethics

Although human subjects research is generally thought to have a high degree of oversight, comparison with animal research illustrates gaps in phase I trial oversight that a more expansive view of procedural ethics can fill. Specifically, the animal research world suggests that there is an important role for facility and staff oversight that could improve healthy‐volunteer trials. For example, unlike for animal research, regulations governing research with humans do not mandate routine inspections of phase I trial sites. The U.S. Food and Drug Administration (FDA), from which drug manufacturers have to obtain approval to commence phase I trials, has the authority to conduct site inspections. However, the division of the agency that could do this is tremendously underresourced and does not have the budget or personnel to do routine safety checks of the tens of thousands of phase I through phase III trial sites under its auspices throughout the country and world.^33^ Besides gaps in facility oversight, clinicians are often not trained for their positions as researchers, receiving little to no instruction on the ethics of research or even good clinical practice guidelines to ensure data are collected appropriately and accurately.^34^ Thus, there are few standards governing phase I facilities or the training of personnel who interact with participants.

Evidence of the potential seriousness of this problem can be found in a particularly illustrative case. The contract research organization SFBC International conducted phase I trials out of a decrepit former hotel until media attention, especially focusing on the company’s recruitment of undocumented immigrants, led to a government investigation and subsequent shutdown of the company’s operations in Miami.^35^ In media coverage of SFBC, it was reported that many clinicians and other research staff members hired to work in phase I research facilities were unlicensed and without research training or experience.

In sum, no oversight body currently has a mandate to ensure trial facilities are safe, clean, and appropriately staffed. Not only are these issues of procedural ethics; they also have direct ties to participants’ welfare. Regulating animal welfare through detailed attention to facility conditions is critical to animal research ethical practices; however, the impact of clinic practices and conditions on healthy volunteers is both highly significant to the volunteers themselves and relatively ignored by ethical analyses of phase I research.

### Translational science quality

Because animal researchers use nonhuman subjects, they are trained to be more reflexive about their selection of model organisms, particularly if the researchers need to justify how their results will translate from their laboratories to human medicine. The use of healthy volunteers in drug development has not generated a parallel imperative to consider translation from healthy participants to affected patients in spite of evidence that phase I participants are not representative of the general population in myriad ways. First, healthy volunteers are predominantly men in their 30s and 40s,^36^ and the literature shows that there are sex‐based differences that could lead to more drug exposure in women than in men.^37^ This is particularly concerning given a government report that drugs that were removed from the market for safety concerns had disproportionately harmful effects on women.^38^ In addition, healthy volunteers are not simply nondiseased adults; rather, they are relatively young individuals who fit quite narrow parameters for blood pressure, metabolic rates, blood counts, and so on. This could mean that these participants provide limited information about how older, ill individuals will tolerate a drug and could mask the scale and scope of adverse drug reactions that might occur after FDA approval. Finally, with the same individuals participating repeatedly in phase I trials, there is also a question about whether the average healthy volunteer has a higher tolerance for investigational drugs than would a random sample of healthy adults. If healthy volunteers suffered frequent uncomfortable adverse effects, it stands to reason that they would be less likely to continue to enroll in these trials. Underscoring the limitations of phase I trial data, a recent study has indicated that safety testing for new pharmaceuticals is woefully inadequate given the number of drugs that are removed from the market or that are given postmarketing safety warnings.^39^

Beyond the selection of participants for phase I trials, the confinement structure of the protocol raises questions about how well results translate to a nonclinical environment. As with animal research, the phase I clinic aims to control as many variables as possible to measure the action of the body on the investigational drug (pharmacokinetics) and the action of the drug on the body (pharmacodynamics). With animal research, this is a reasonable mechanism to design and conduct research on organisms that could not otherwise be used in science. With human participants, however, it is less clear how and why all trial restrictions are necessary, particularly when they create an artificiality that will never be duplicated once a drug is widely available. If these protocol choices impinge on the translation of trial results, it is all the more problematic that healthy volunteers are subjected to restrictions that could diminish their welfare.

In general, there needs to be more explicit recognition of the limitations of trial results when phase I trials are conducted on healthy volunteers in confinement. Indeed, the nonrepresentativeness of healthy volunteers coupled with the confinement structure raises questions about whether phase I trials should, in fact, be conducted as they typically are. Returning to the principle of beneficence, the translational science question is paramount because healthy volunteers should not be exposed to even minimal risk if the results of the research have limited benefit in providing information about the safety and tolerability of drugs in affected patients. This line of reasoning implies that concern about translation should be a central part of the science‐value assessment for phase I trials when considering whether a protocol is ethical. For instance, while the use of healthy volunteers and a restrictive confinement structure is normative for phase I trials, researchers should instead be required to provide a stronger scientific rationale for this type of study design. Importantly, it is the model‐organism lens that helps to raise these kinds of translational science questions that are critical for both phase I healthy‐volunteer and nonhuman‐animal studies.

### Respect and justice for healthy volunteers

So far, we have reimagined concerns of beneficence, procedural ethics, and science value through concepts and mechanisms offered within the animal research ethics and policy framework. While we neither suggest that those concepts and mechanisms are adequate in the animal subject case nor argue that they be transported wholesale to human subjects, we assert that this comparative work helpfully shifts perspective toward workable solutions to the unique ethical and policy problems of phase I healthy‐volunteer research. Of course, there is no direct analog for respect for persons and substantive justice in conventional animal research oversight, but that does not mean that such concepts are inapplicable to a proper ethics of the use of model organisms even in animal research.

At the same time, the insight that serial healthy volunteers, like model organisms, are standardized across diverse trial protocols and readily available for the pharmaceutical industry serves as a reminder that a focus on questions of respect and justice for this population requires a novel framing. First, welfare is deeply concordant with the principle of respect for persons. Making promotion of welfare an ethical requirement pushes respect for persons beyond the narrow focus on simply obtaining valid informed consent. Indeed, this approach highlights the importance of revising the conventional interpretation of respect for participant autonomy to include the physical environment of the clinic and the respectful treatment of participants as persons during confinement.

Second, the principle of justice would be enhanced by a change in procedural ethics oversight requiring inspections of phase I trial sites and staff training. The clinics that underinvest in their infrastructure and other resources contribute to potentially exploiting individuals who have the most critical financial need. It is not a coincidence that some of the most vulnerable members of society, undocumented immigrants, were the primary group of participants at the unsafe SFBC facility that was shuttered after media and government scrutiny.

Finally, to further avoid exploitation and promote the just treatment of phase I trial participants, careful attention must be paid to how healthy volunteers can become, in some sense, career model organisms. Because the time commitment for such trials is difficult to square with regular full‐time employment and serial participation does not constitute a work history, the more financially vulnerable participants may feel trapped within a cycle of repeat enrollment in trials. While phase I participation thereby essentially becomes their job, current ethical oversight mechanisms, which insist on viewing compensation as payment merely for time and inconvenience, often fall short of protecting participants. Arguably, then, these participants who have become so readily available to the pharmaceutical industry deserve at least worker‐status protections.^40^ Thus, the model‐organism framework can enhance the protection of healthy volunteers even in arenas not traditionally addressed (whether rightly or wrongly) within animal research ethics and policy by integrating concerns for the confinement structure and serial nature of participation in healthy‐volunteer phase I trials.

## VALUE OF THE MODEL‐ORGANISM FRAMEWORK FOR HEALTHY‐VOLUNTEER TRIALS

**R**esearch scientists, regulatory personnel, industry representatives, and bioethics scholars tend to focus their work and interests on either human or animal subjects but, outside of concerns about extrapolation from animal studies to human research, rarely consider a comparative approach to these research arenas. While animal studies have their own host of ethical concerns, a model‐organism framework nonetheless stimulates new analyses of the specialized ethics and policy considerations surrounding the use of healthy volunteers in drug development. In spite of the unique structural features of healthy‐volunteer trials, there is currently no guidance specific to this type of research, and ethics and policy standards developed for other trial contexts are insufficient and poorly tailored to phase I research.

The model‐organism framework is a useful source of guidance in responding to important ethics and policy gaps in phase I trials. For example, serial participation raises significant questions about exploitation of minority groups in the unfair distributions of the benefits and burdens of research participation, as well as the potential for their undue inducement to participate against a backdrop of economic vulnerability. The model‐organism framework we propose here does not “solve” the problem of undue inducement in phase I research or rectify the overrepresentation of minorities as healthy volunteers. Even so, it does recast respect for persons, beneficence, and justice in a new light as well as bolster procedural ethics and the translational science value of phase I trials. We have shown, as a practical matter, how participant confinement and monitoring during the study period invite questions of how to regulate the research environment, respectfully engage with participants, and provide for their comfort and welfare. We have emphasized, moreover, how the model‐organism framework reveals the specific need for focus on the translational science‐value questions little recognized in phase I studies as typically designed and conducted. Together, these recommendations offer serious promise of a payoff in improved ethics and policy oversight of phase I healthy‐volunteer clinical trials through advancement of the model‐organism framework we propose.

## ACKNOWLEDGMENT

Research reported in this article was supported by the National Institute of General Medical Sciences of the National Institutes of Health under award number R01GM099952.
